# Selenium-Stimulated Exosomes Enhance Wound Healing by Modulating Inflammation and Angiogenesis

**DOI:** 10.3390/ijms231911543

**Published:** 2022-09-29

**Authors:** June Seok Heo

**Affiliations:** Cell Therapy Center, Severance Hospital, Seoul 03722, Korea; juneseok@yuhs.ac; Tel.: +82-2-2228-7822

**Keywords:** angiogenesis, exosome, inflammation, mesenchymal stem cell, selenium, wound healing

## Abstract

Mesenchymal stem cell (MSC)-derived exosomes have emerged as an attractive cell-free tool in tissue engineering and regenerative medicine. The current study aimed to examine the anti-inflammatory, pro-angiogenic, and wound-repair effects of both exosomes and selenium-stimulated exosomes, and check whether the latter had superior wound healing capacity over others. The cellular and molecular network of exosomes, as a paracrine signal, was extensively studied by performing miRNA arrays to explore the key mediators of exosomes in wound healing. Selenium is known to play a critical role in enhancing the proliferation, multi-potency, and anti-inflammatory effects of MSCs. Selenium-stimulated exosomes showed significant effects in inhibiting inflammation and improving pro-angiogenesis in human umbilical vein endothelial cells. Cell growth and the migration of human dermal fibroblasts and wound regeneration were more enhanced in the selenium-stimulated exosome group than in the selenium and exosome groups, thereby further promoting the wound healing in vivo. Taken together, selenium was found to augment the therapeutic effects of adipose MSC-derived exosomes in tissue regeneration. We concluded that selenium may be considered a vital agent for wound healing in stem cell-based cell-free therapies.

## 1. Introduction

Wound healing refers to the dynamic cellular and molecular events that occur in damaged or destroyed tissues, including inflammation, cell growth and migration, angiogenesis and vascularization, and tissue remodeling [1,2]. Since wounds involve highly complex biological processes, they can progress to delayed-healing or chronic non-healing wounds with ineffective therapies [3]. Therefore, optimal comprehensive methods have been developed for more effective and innovative therapies. For instance, adipose-derived mesenchymal stem cells (AdMSCs) have been proven to enhance the process of wound healing by regulating the immune response, promoting angiogenesis, and accelerating epithelialization [4].

Mesenchymal stem cells obtained from adipose tissue, bone marrow, cord blood, and placenta are attractive sources since they are free from ethical and safety problems. Despite the advantages, MSCs have sometimes been reported to undergo chromosomal abnormalities and form malignant tumors after transplantation [5]. In addition, Toma et al. found that a proportion of surviving MSCs decrease in clinical investigations [6]. These findings suggested that paracrine effects, including growth factors, cytokines, chemokines, bioactive lipids, or microvesicles, may contribute to most of the positive results obtained using MSCs [7]. In particular, MSC-derived exosomes have recently attracted considerable attention in tissue engineering owing to their contribution in the healing of damaged or injured tissues and organs by transferring miRNAs, mRNA molecules, peptides, proteins, cytokines, and lipids originating from the MSCs [8]. Exosomes derived from MSCs can act in a paracrine manner, and have been extensively applied in the stem cell-based research of many diseases [9]. Previous studies have supported the use of exosomes in the establishment of a cell-free therapeutic approach for a safer therapeutic modality in a variety of diseases [10].

Exosomes secreted by cells are nano-sized (30–150 nm) small membrane-bound extracellular vesicles that carry bioactive molecules, such as nucleic acids, proteins, and lipids; they play a major role in intercellular communication. In other words, exosomes modulate physiological and pathological processes by transferring key signaling biomolecules to specific recipients and their surrounding cells [11,12]. Exosomes can promote proliferation, migration, and angiogenesis in wound processes when transplanted into animal wound models, thereby proving to be promising therapeutic tools for wound repair [13,14]. In agreement with the above findings, AdMSC-derived exosomes have recently been shown to repair wounds by modulating inflammation and angiogenesis [15]. Nevertheless, several challenges, such as the in vitro expansion of MSCs without senescence and the large-scale production of exosomes to enhance their paracrine actions, still remain to be overcome [16].

The optimization of MSC culture conditions using cytokines, hypoxia, trophic factors, physical factors, and chemical and pharmacological agents is one of the key strategies for improving MSC function in regenerative medicine [17,18]. Several recent studies have demonstrated that MSCs stimulated by beneficial substances exert enhanced paracrine effects [19]. For example, the preconditioning of MSCs with polyribonucleic acid improved their therapeutic efficacy in polynitrotriphenylsulfonate-induced colitis [20]. Based on such results, preconditioning with various physical, chemical, and biological factors has been applied to enhance exosome functions. Various studies have shown that exosomes stimulated by deferoxamine facilitate cutaneous wound healing by activating angiogenesis [21]. Moreover, melatonin-stimulated MSC-derived exosomes were reported to enhance wound healing by regulating macrophages [22].

In general, selenium is necessary for cellular function in humans and is a well-known antioxidant and cofactor of many enzymes that inhibit oxidation and inflammation [23]. Previous studies have demonstrated that selenium can promote stem cell proliferation and improve the multipotency of AdMSCs [24]. For instance, Park et al. has shown that selenium improves the therapeutic effects on tissue repair through the paracrine action of MSCs, suggesting that preconditioning AdMSCs with selenium may enhance the effects of AdMSC-derived exosomes on wound healing [25]. Thus, in this study, we examined whether exosomes derived from selenium-stimulated AdMSCs could enhance wound healing by modulating inflammation and angiogenesis. The effect of selenium-stimulated exosomes on the wound healing process, including inflammation and angiogenesis, was compared to that of selenium and exosomes alone. To understand the mechanisms better, miRNA arrays were performed, results for exosomes and selenium-stimulated exosomes were compared, and related cellular and molecular studies were conducted using in vitro and in vivo wound healing models.

## 2. Results

### 2.1. Selenium Treatment Improved AdMSC Functionality

**Figure 1:**
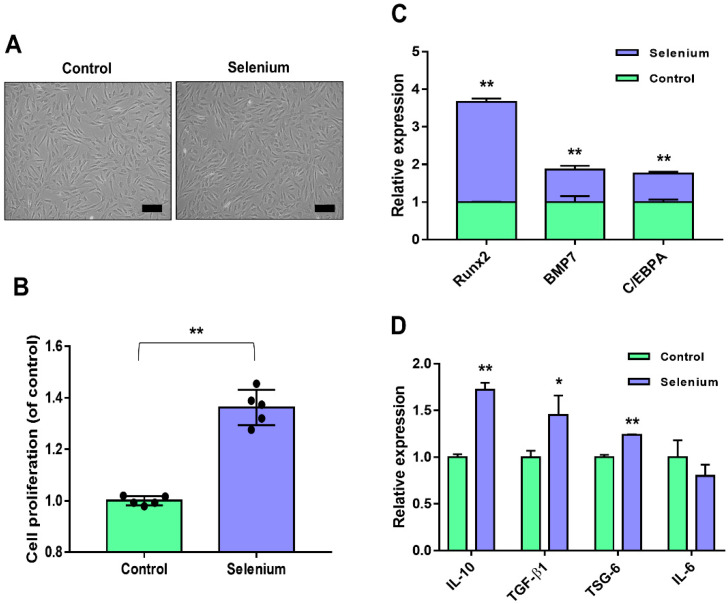
Effects of selenium on the stemness of AdMSCs. (**A**) A picture of the cell morphology was taken before and after treatment with 5 ng/mL selenium (100× magnification, scale bar = 200 μm). (**B**) Proliferation rates of AdMSCs after exposure to selenium were determined using a WST-based assay. (**C**) Relative mRNA levels of key genes for multi-lineage differentiation were analyzed by real-time PCR. (**D**) Relative mRNA levels of inflammation-related genes were evaluated by real-time PCR. Data are expressed as the mean ± SD of three independent experiments. * Significant difference from control, *p* < 0.05. ** Significant difference from control, *p* < 0.01.

The applied concentration (5 ng/mL) of selenium was based on previously reported culture conditions of stem cells [25]. Figure 1A shows the preconditioning effect of selenium on the morphology of AdMSCs during culture. Selenium-treated AdMSCs displayed a morphology similar to that of typical spindle-shaped MSCs derived from untreated cells (Figure 1A). To examine the effect of selenium on the proliferation activity of AdMSCs, cell growth was evaluated using water-soluble tetrazolium salt. The proliferation rates of AdMSCs were remarkably increased under the selenium-treated conditions (Figure 1B).

To assess whether selenium affected the differentiation potential of AdMSCs, the mRNA expression levels of key transcription factors of multipotency were analyzed. The expression levels of runt-related transcription factor 2 (Runx2) for osteogenesis, bone morphogenic protein 7 (BMP7) for chondrogenesis, and CCAAT enhancer binding protein α (C/EBPA) for angiogenesis were significantly enhanced in the selenium-treated AdMSCs (Figure 1C). To test the anti-inflammatory potential of AdMSCs, inflammation-related mRNA levels were investigated using real-time PCR. The mRNA levels of IL-10, TGF-β1, and TSG-6 (anti-inflammatory genes) were elevated in AdMSCs cultured with selenium, whereas the expression level of IL-6 (associated with inflammation) was decreased compared to that in untreated cells although there was no significance (Figure 1D). Taken together, the results indicated that pretreatment of AdMSCs with selenium enhanced their functions, including the proliferative activity, differentiation potential, and anti-inflammatory capacity.

### 2.2. AdMSC-Derived Exosomes and Selenium-Stimulated Exosomes Showed Similar Characteristics

**Figure 2:**
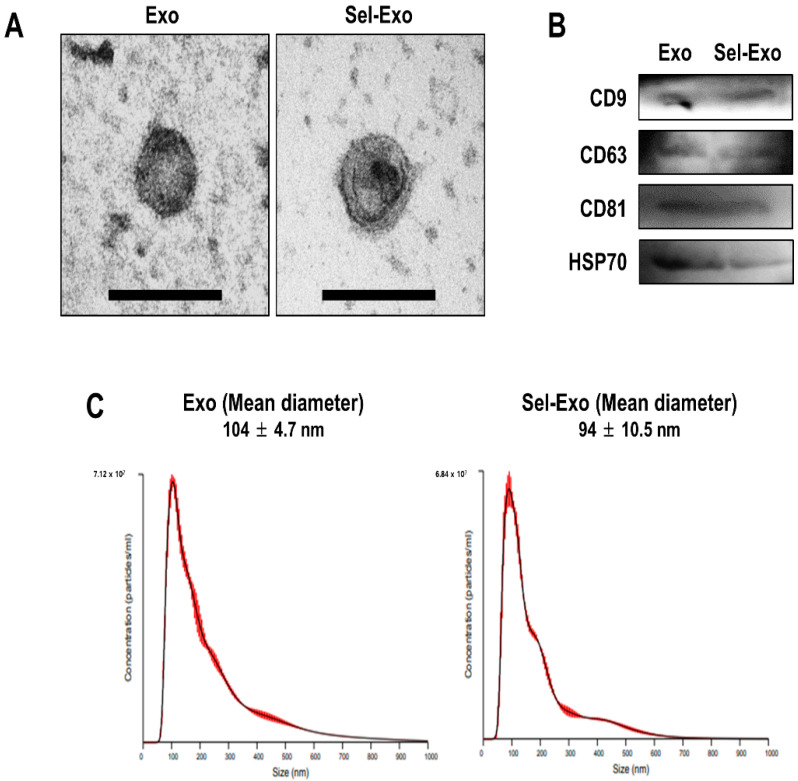
Characterization of exosomes (Exo) and selenium-treated exosomes (Sel-Exo) derived from AdMSCs. (**A**) Morphology of Exo and Sel-Exo was analyzed by transmission electron microscopy (TEM) (scale bar = 200 nm). (**B**) The exosomal markers (CD9, CD63, CD81, and HSP70) for Exo and Sel-Exo were evaluated by Western blotting. (**C**) The mean size of the particles for Exo and Sel-Exo was measured by nanoparticle tracking analysis (NTA).

Exosomes were successfully isolated from the supernatants of AdMSCs treated with or without selenium. To verify the morphology of the exosomes (Exo) and selenium-treated exosomes (Sel-Exo), transmission electron microscopy (TEM) was used. The results showed that both types of exosomes were cup-shaped membrane-bound vesicles, with no significant difference between them (Figure 2A). Subsequently, Western blotting was performed to identify the exosomal markers in both cell types. CD9, CD63, CD81, and HSP 70, specific exosomal proteins, were detected in both Exo and Sel-Exo, showing no significant difference between them (Figure 2B, Figure S1). In addition, nanoparticle tracking analysis (NTA) revealed that the size of the Exo and Sel-Exo ranged from 30 nm to 150 nm, with mean diameters of 104 nm and 94 nm, respectively (Figure 2C). Collectively, the results showed no significant difference between Exo and Sel-Exo.

### 2.3. Selenium-Treated Exosomes Strongly Promoted Cell Proliferation by Reducing p16, p21, and Intracellular ROS Accumulation

**Figure 3:**
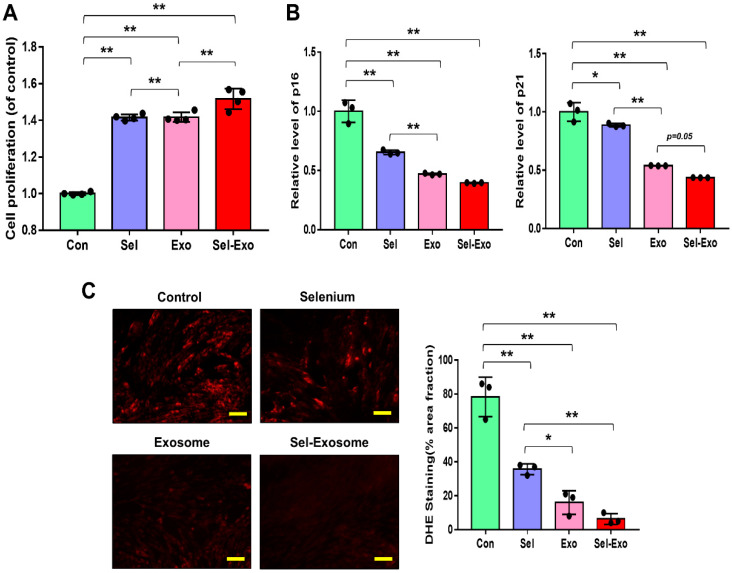
Proliferation activity of AdMSCs treated with selenium, exosomes, and selenium-treated exosomes. (**A**) The proliferation rates were determined after exposure to selenium, exosomes, and selenium-treated exosomes. (**B**) The mRNA expression levels of senescence-related p16 and p21 were evaluated by real-time PCR. (**C**) ROS levels were determined by dihydroethidium (DHE) staining using fluorescence microscopy (scale bar = 100 μm). A representative image of three independent experiments is shown. Data are expressed as the mean ± SD of three independent experiments. * Significant difference from control, *p* < 0.05. ** Significant difference from control, *p* < 0.01.

To confirm the effect of selenium-treated exosomes (Sel-Exo), we first compared the proliferation rates of AdMSCs cultured with 5 ng/mL selenium, 5 μg/mL exosomes, and 5 ng/mL selenium-treated 5 μg/mL exosomes. The highest proliferation rates were observed in AdMSCs cultured with Sel-Exo (Figure 3A). Next, we analyzed the expression levels of p16 and p21 to investigate the cellular senescence-related molecular changes. As shown in Figure 3B, the mRNA levels of p16 and p21 were reduced in all cells cultured with Se, Exo, and Sel-Exo. The cells cultured with Exo and Sel-Exo showed no remarkable difference in real-time PCR analysis while displaying the lowest expression levels in Sel-Exo (Figure 3B). To further examine the changes in the growth rate upon Sel-Exo treatment, intercellular reactive oxygen species (ROS) levels, the most critical factor in cell senescence, were observed using a fluorescence microscope. As shown in Figure 3C, significant differences in ROS levels were observed in cells treated with Se, Exo, and Sel-Exo. In particular, Sel-Exo treatment resulted in the greatest decrease in ROS levels (Figure 3C). Collectively, the findings demonstrated that Sel-Exo improves the growth activity of AdMSCs through the inhibition of senescence-related p16, p21, and ROS levels.

### 2.4. Selenium-Treated Exosomes Suppressed the Inflammatory Response by Regulating Pro-Inflammatory and Anti-Inflammatory Factors

**Figure 4:**
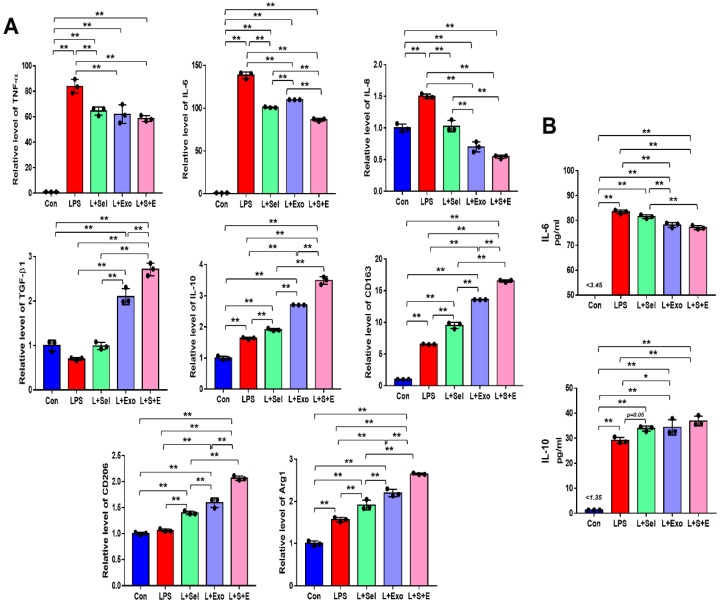
Effects of selenium, exosomes, and selenium-treated exosomes on the inflammatory response. (**A**) Relative gene expression related to pro-inflammation (TNF-α, IL-6, and IL-8) and anti-inflammation (TGF-β1 and IL-10) was evaluated by real-time PCR after LPS treatment. Relative gene expression levels of CD163, CD206, and Arg1 were detected. (**B**) A multiplex assay was conducted to detect the concentrations of IL-6 and IL-10 in the conditioned media. Data are expressed as the mean ± SD of three independent experiments. * Significant difference from control, *p* < 0.05. ** Significant difference from control, *p* < 0.01.

To evaluate the effect of selenium-treated exosomes on the inflammatory response, THP-1 (a human monocytic cell line derived from an acute monocytic leukemia patient) cells were treated with 100 ng/mL lipopolysaccharide (LPS). After 1 day, real-time PCR was performed to evaluate the relative expression of pro-inflammatory and anti-inflammatory genes. Overall, the results indicated that the relative levels of pro-inflammatory genes (TNF-α, IL-6, and IL-8) in the selenium, Exo, and Sel-Exo groups were decreased, whereas those of anti-inflammatory genes (TGF-β1 and IL-10) were elevated in the treated groups (Figure 4A). In particular, we noticed IL-6 expression in the Sel-Exo group to be significantly decreased than that in the selenium and Exo groups, and TGF-β1 and IL-10 expression in the Sel-Exo group to be remarkably increased than in the other groups (Figure 4A). In addition, we noticed that the relative levels of anti-inflammatory M2 macrophage markers (CD163, CD206, and Arg1) in the Sel-Exo group were significantly enhanced compared to those in the other groups, illustrating that M2 macrophages increased by Sel-Exo could promote anti-inflammatory effects. Using multiplex assays, we confirmed the secretion of IL-6 in the Sel-Exo group to be remarkably decreased compared to that in the LPS-treated group while that of IL-10 in the Sel-Exo group was remarkably increased compared to that in the LPS-treated group (Figure 4B). The results indicated that Sel-Exo can enhance the anti-inflammatory effect by regulating inflammation-associated genes and immune cells.

### 2.5. Selenium-Treated Exosomes Accelerated Endothelial Cell Angiogenesis In Vitro

**Figure 5:**
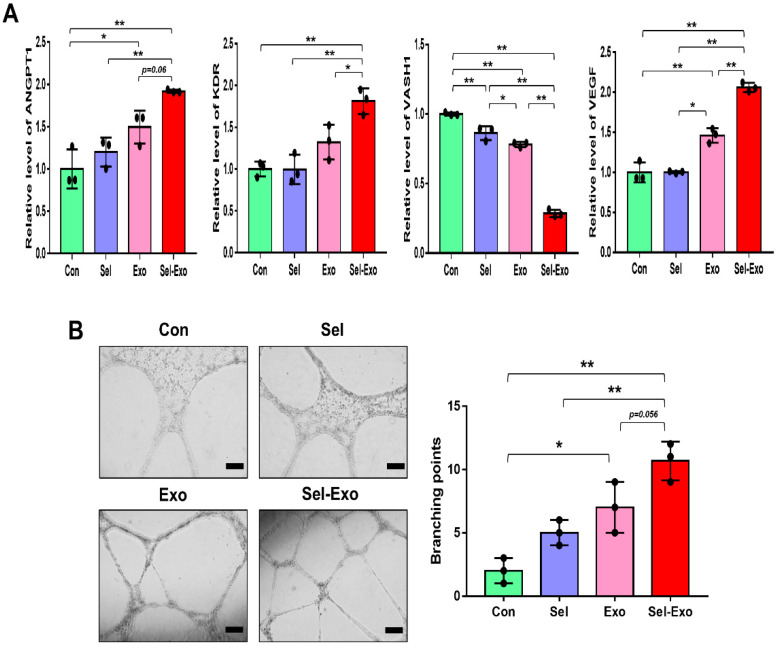
Effects of selenium, exosomes, and selenium-treated exosomes on angiogenic potential in vitro. (**A**) The expression levels of pro-angiogenic and anti-angiogenic mRNAs were evaluated by real-time PCR. (**B**) An in vitro tube formation assay was performed using Matrigel. Tube-like structures were observed using an inverted microscope (100× magnification, scale bar = 200 μm). A representative image of three independent experiments is shown. Data are expressed as the mean ± SD of three independent experiments. * Significant difference from control, *p* < 0.05. ** Significant difference from control, *p* < 0.01.

To investigate the effect of Sel-Exo on angiogenesis, HUVEC (Human Umbilical Vein Endothelial Cells) were used. The expression levels of pro-angiogenic genes, angiopoietin1 (ANGPT1) and flk1 (KDR), and of the anti-angiogenic gene, vasohibin-1 (VASH1), were analyzed by real-time PCR. We noticed the expression levels of ANGPT1, KDR and VEGF to be significantly enhanced in the Sel-Exo group than in the other groups, whereas the expression of VAHS1 was remarkably reduced in the Sel-Exo group, indicating that Sel-Exo could promote angiogenesis (Figure 5A). Based on the real-time PCR results, we carried out a tube formation assay to further investigate the effects on angiogenesis in vitro. As shown in Figure 5B, tube formation in the Sel-Exo group was significantly enhanced compared to that in the other groups. Taken together, the results demonstrated that Sel-Exo strongly improved the in vitro angiogenic potential by regulating both pro-angiogenic and anti-angiogenic genes.

### 2.6. Selenium-Treated Exosomes Promoted Migration and Wound Closure by Upregulating the Factors Associated with Remodelling

**Figure 6:**
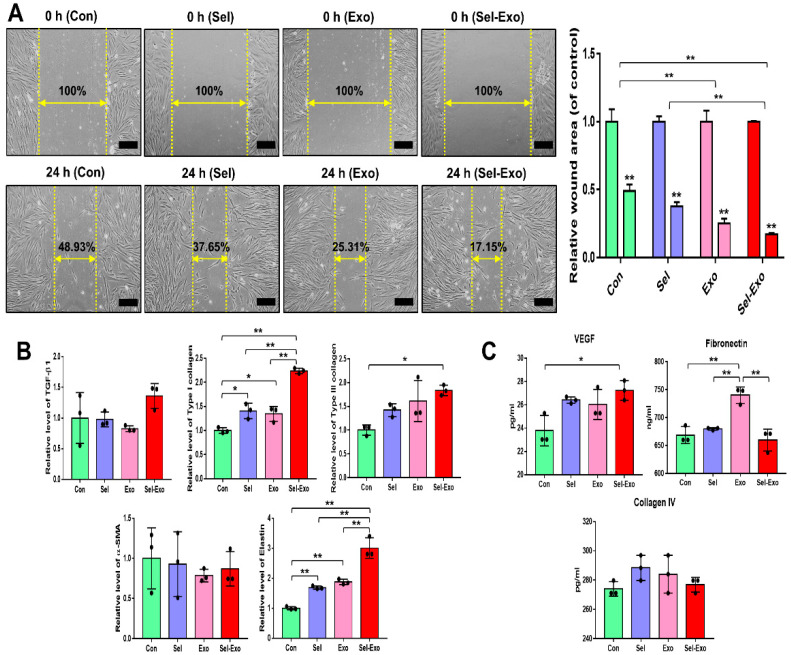
Migration of human dermal fibroblasts in the presence of selenium, exosomes, and selenium-treated exosomes. (**A**) Optical images of migrated fibroblasts were obtained from the in vitro scratch-wound-closure assay, and the relative migration rates of the fibroblasts were quantified (100× magnification, scale bar = 200 μm). (**B**) Relative mRNA expression levels were evaluated by real-time PCR. (**C**) Relative protein levels of the corresponding beneficial factors for wound healing were determined by multiplex assay. A representative image of three independent experiments is shown. Data are expressed as the mean ± SD of three independent experiments. * Significant difference from control, *p* < 0.05. ** Significant difference from control, *p* < 0.01.

To investigate whether Sel-Exo enhanced migration, the migration of dermal fibroblasts was assessed using an in vitro scratch assay. Figure 6A clearly shows that cells in the Sel-Exo group migrated significantly faster for wound closure than those in the other groups. Overall, relatively higher expression levels of migration- and remodeling-related factors, such as TGF-β1, type I collagen, type III collagen, alpha smooth muscle actin (α-SMA), and elastin, were found in the Sel-Exo group (Figure 6B). In particular, the expression of type I collagen and elastin for wound healing was remarkably elevated in the Sel-Exo group than in the others (Figure 6B). We also found the concentration of vascular endothelial growth factor (VEGF), which stimulates wound healing, to be remarkably higher in the Sel-Exo group than in the others (Figure 6C). Contrary to expectations, high expression levels of fibronectin were suppressed by the selenium treatment. Various fibronectin types play many different roles in wound healing by interacting with different cell types, cytokines, and chemicals. In our results, selenium may have different effects on fibronectin receptors (Figure 6C). Different types and organization of collagens are involved in wound healing. Collagen IV is known as a mediator of inflammation and its wound healing was analyzed, however there were no significance (Figure 6C). The results collectively suggested that in vitro cell migration for wound closure was improved by the active migration of fibroblasts, as well as by the enhanced production of beneficial factors that are required in cell proliferation and migration, remodeling, and the extracellular matrix (ECM) required for wound healing.

### 2.7. Selenium-Treated Exosomes Contained Many Beneficial miRNAs for Wound Healing

**Figure 7:**
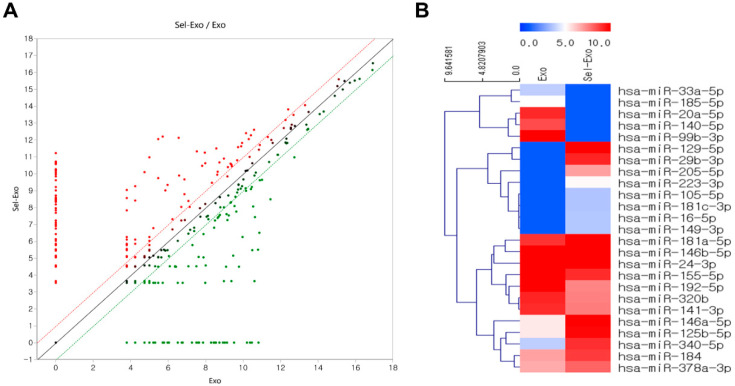
Distribution of differentially expressed selenium-treated exosomes compared to exosomes. (**A**) Scatter plots show the differential miRNA expression between two samples. (**B**) Hierarchical clustering analysis indicates organized miRNA expression in wound repair.

Exosome small RNA sequencing was conducted to explore the upregulated or downregulated miRNAs related to cell proliferation and migration, inflammation, angiogenesis, and remodeling for wound repair, with Exo as the sample and Sel-Exo as the test sample. The fold-change was analyzed by dividing the normalized expression profile of Sel-Exo by that of the corresponding Exo. Surprisingly, the scatter plots of miRNA expression were significantly different between Exo and Sel-Exo (Figure 7A). As described previously [26], extensive miRNA data were focused on four categories (cell proliferation and migration, inflammation, angiogenesis, and remodeling) based on the biological processes involved in wound regeneration to reduce the complexity of the analysis. The clustering heatmap in Figure 7B shows miRNAs that were significantly different between the two samples. Upregulated miRNAs included miR-146a-5p, miR-340-5p, miR-223-3p, miR-125b-5p, miR-16-5p, miR-149-3p, miR-105-5p, miR-181c-3p, miR-146b-5p, and miR-181a-5p in the anti-inflammatory category; miR-378a-3p in the pro-angiogenic category; miR-205-5p, miR-146a-5p, and miR-184 in the proliferation and migration category; and miR-129-5p and miR-29b-3p in the remodeling category. Downregulated miRNAs included miR-140-5p, miR-33a-5p, and miR-155-5p in the pro-inflammatory category; miR-20a-5p, miR-192-5p, miR-24-3p, and miR-320b in the anti-angiogenic category; and miR-99b-3p, miR-185-5p, and miR-141-3p in the anti-proliferation and migration category [27,28,29,30,31,32]. The upregulation and downregulation of significant miRNAs between the Exo and Sel-Exo groups are listed in Tables S2 and S3. Moreover, gene ontology enrichment of the top 10 upregulated miRNAs focused on biological processes has been summarized in Figure S2.

### 2.8. Selenium-Treated Exosomes Improved Wound Healing In Vivo

**Figure 8:**
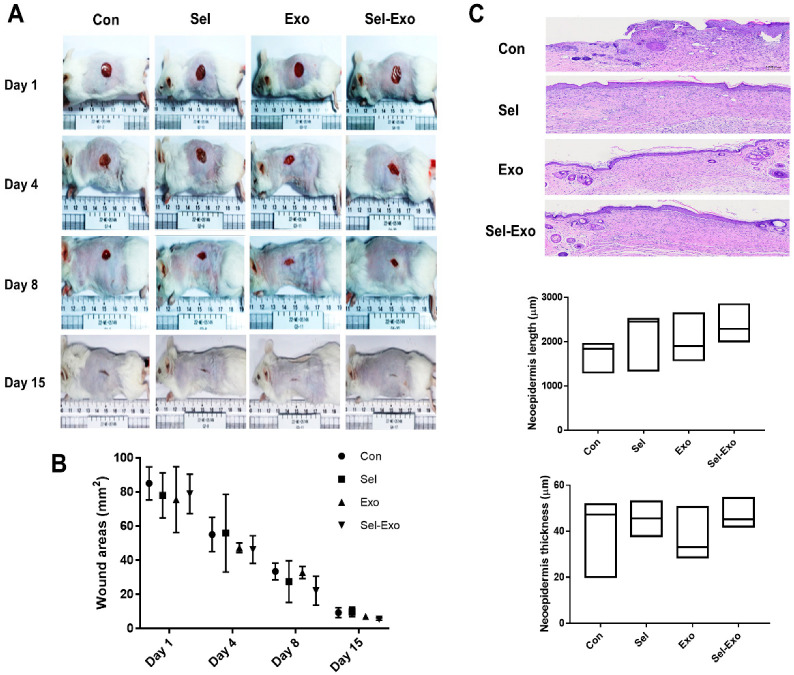
In vivo wound healing effect. (**A**) Optical images were obtained from the control group, selenium group, exosome group, and selenium-treated exosome group at days 1, 4, 8, and 15 (scale = cm). (**B**) Quantitative analysis, based on optical images, was performed on the wound area. (**C**) H&E-stained images of tissues and related quantification of neoepidermis length and neoepidermis thickness were conducted in the control group, selenium group, exosome group, and selenium-treated exosome group at day 15 (5 male donors from each group) (scale bar = 300 μm).

To further explore the effect of Sel-Exo on wound healing, an in vivo cutaneous wound healing assay was performed using ICR mice. Optical images of wounds treated with PBS, selenium, Exo or Sel-Exo were observed for 15 days. Equal volumes (100 μL) were subcutaneously injected into the mice, and the PBS-injected group was used as a control. All groups showed wound closure after 15 days in the optical images, without a significant difference in the wound area (Figure 8A). Notably, quantitative analysis showed that treatment with Sel-Exo resulted in enhanced wound repair compared to that in the other groups; however, the difference was not significant (Figure 8B). Among the groups, Sel-Exo showed the greatest increase in length and thickness of the neoepidermis, although the difference was not significant (Figure 8C). These results could possibly be attributed to the accelerated growth and migration of dermal fibroblasts along with upregulated beneficial factors for wound healing, as shown in Figure 6. Taken together, the findings confirmed the possibility of accelerated wound healing due to treatment with Sel-Exo derived from AdMSCs.

## 3. Discussion

Accumulating evidence has shown that microvesicles secreted from MSCs can be used in stem cell-based therapies [33]. Previously, we had demonstrated that exosomes derived from AdMSCs expedite wound healing by modulating inflammation and angiogenesis. In addition, the molecular mechanism via which exosomes mediate wound healing had been reported [15,26]. In this study, we suggested the following three categories of stem cell therapy: first-generation stem cell therapy, which involves direct or differentiated stem cell transplantation; second-generation stem cell therapy that uses microvesicles, such as exosomes, which are stem cell-based cell-free transplants; and third-generation stem cell therapy that involves the transplantation of exosomes with enhanced functionality.

Wound healing is a complex biological process related to a variety of cells, cellular events, molecular events, and multiple phases of immune cells, such as macrophages for inflammation, fibroblasts for proliferation and migration, and endothelial cells for angiogenesis. In particular, the inflammatory response and angiogenesis play major roles in wound healing, because delayed inflammation and angiogenesis have adverse effects on the subsequent repair and can even develop into chronic wounds [34,35]. Exosomes pretreated with various substances can exert diverse effects on cell-to-cell or cell-to-microenvironment communication. Hence, at first, we aimed to explore a chemical element with anti-inflammatory and pro-angiogenic effects along with wound healing effects. Previously, Park et al. had reported that antioxidant selenium should be used as an essential supplement for tissue repair and regeneration. They demonstrated that selenium can improve in vitro expansion and preserve the stemness of MSCs, resulting in therapeutic effects on wound healing [25]. Based on their results, we hypothesized that selenium treatment could endow AdMSC-derived exosomes with improved biological effects, reducing inflammation, and enhancing angiogenesis; thereby promoting wound healing. Accordingly, selenium has been applied to produce enhanced exosomes for wound repair. In our system, selenium increased the cell proliferation rate without any morphological change. Moreover, at the molecular level, selenium treatment resulted in elevated expression of key multipotency markers and anti-inflammatory mRNAs compared to that in the control, implying that selenium induces positive changes in AdMSCs, which is in agreement with a previous study [25]. Subsequently, we produced selenium-primed exosomes from AdMSCs for applying to multiple processes for wound healing. Interestingly, we found that exosomes treated with selenium strongly facilitated cell proliferation by inhibiting *p16* and *p21* mRNA expression. Mitochondrial dysfunction triggered by ROS induces replicative senescence, which arrests growth due to oxidative stress accumulation in MSCs [36]. To understand the effects of selenium-treated exosomes on cell growth and *p16* and *p21* molecular levels, we analyzed the ROS levels in cells. The results clearly indicated that selenium-treated exosomes can significantly increase the growth rate of AdMSCs by inactivating *p16* and *p21* at the mRNA level and inhibiting ROS accumulation, implying that selenium in antioxidants reinforces exosomes as ROS inhibitors that are involved in cellular senescence. Previous studies have shown that anti-inflammatory mRNAs are enhanced during exosome treatment through the inhibition of pro-inflammatory mRNAs [15]. In the present study, selenium-treated exosomes increased M2 macrophage-related markers in an LPS-induced environment by decreasing M1 macrophage-related markers, leading to an anti-inflammatory microenvironment. M2 macrophage polarization in the wound healing process helps vascular conditions by promoting pro-angiogenesis and extracellular matrix (ECM) synthesis [37]. The enhanced anti-inflammatory effects of selenium-treated exosomes were supported by enzyme-linked immunosorbent assay (ELISA) analysis at the protein level, including IL-6 and IL-10. Unfortunately, significant findings were not obtained because the untreated exosomes have an impact on inflammatory responses. The unsatisfactory results might be a consequence of the selenium doses. Importantly, the present study clearly confirmed the possibility that the production of exosomes from AdMSCs after pretreatment with selenium can result in more effective results. Poor angiogenesis is regarded as an important cause of delayed wound healing [38]. With respect to the angiogenic effects, the current study showed that the pro-angiogenic genes *ANGPT1 (angiopoietin 1)*, *KDR (flk1)* and *VEGF (Vascular Endothelial Growth Factor)* were significantly enhanced, whereas the anti-angiogenic gene *VASH1 (vasohibin-1)* was remarkably reduced in the selenium-treated exosome group. The results showed significantly enhanced in vitro tube formation (angiogenesis) in HUVECs due to selenium-treated exosomes, implying that selenium strengthens exosomes as an angiogenic enhancer. Furthermore, in the in vitro scratch wound model, the wounds closed the fastest in the selenium-treated exosomes. The results were supported by the elevation of type I collagen, elastin, and VEGF as tissue regeneration-related factors. Other factors involved in tissue remodeling, except for those with significant differences, should be further analyzed, focusing on the activation or inactivation of other signaling molecules. Taken together, the positive results were indicative of both the anti-inflammatory and synergic pro-angiogenic functions of selenium-stimulated AdMSC-derived exosomes in wound healing.

Generally, exosomes, as messengers, deliver proteins, cytokines, growth factors, DNA, mRNA, and miRNA from originating cells to target cells, resulting in various cellular and/or molecular changes in the recipient cells. We confirmed that selenium-treated exosomes contained effective miRNAs that are related to cell proliferation and migration, anti-inflammation, pro-angiogenesis, and remodeling for wound repair compared to untreated exosomes. The gain and loss of miRNAs in exosomes secreted by AdMSCs and selenium-treated AdMSCs are listed in Tables S2 and S3, respectively. Although some miRNAs should be validated, the primary aim of this study was to explore the candidate miRNAs that are associated with wound regeneration. Based on the present data, we plan to further evaluate miRNAs and explore the downstream pathways related to selenium-treated miRNAs for wound repair, in future. Unfortunately, significant findings were not obtained, although selenium-treated exosomes were applied to the experimental animals. The unsatisfactory results might be a consequence of the high inter-animal variation, along with a failure to provide the optimal selenium or exosome doses required for efficient results with selenium-treated exosomes. However, the histopathological analysis revealed that selenium-treated exosomes promote epidermalization by affecting the length and thickness of the new epidermis.

In conclusion, the present data suggested that selenium-treated exosomes may exert superior effects on wound healing. It further suggested that selenium could be used to enhance exosome activity, reduce inflammation, improve proliferation and migration, and enhance angiogenesis, ultimately resulting in wound healing. In other words, selenium could be a trigger for enhanced therapeutic potential in next-generation stem cell-based cell-free therapeutic applications. The results might provide insight into safer and more efficient stem cell therapies using advanced exosomes and contribute to a better understanding of stem cell biology. Further investigations to identify novel chemical elements and optimal doses of exosomes for wound healing will be undertaken in future.

## 4. Materials and Methods

### 4.1. Cell Culture

Human adipose tissue-derived mesenchymal stem cells (AdMSCs) and human umbilical vein endothelial cells (HUVECs) were purchased from CEFO (www.cefobio.com), Seoul, Republic of Korea. Human dermal fibroblasts were obtained from Invitrogen (Carlsbad, CA, USA). The cells were cultivated in culture medium (DMEM-low glucose containing 10% FBS, 100 U/mL penicillin, and 100 µg/mL streptomycin) in a 75-cm^2^ culture flask (Nunc, Roskilde, Denmark) at 37 °C in a 5% CO_2_-containing atmosphere. They were subcultured in a 175-cm^2^ culture flask (Nunc) for expansion after they had reached approximately 90% confluence. The medium was changed every three or four days. For selenium treatment, AdMSCs were cultured with 5 ng/mL sodium selenite (Sigma Chemical Co., St. Louis, MO, USA). The cells obtained from 3 donors were used for the present study. For the inflammatory response, THP-1 cells, a human monocytic cell line derived from an acute monocytic leukemia patient were used. The THP-1 cells were cultured and maintained in RPMI medium supplemented with 10% FBS and 1% P/S at 37 °C in a 5% CO_2_ environment (all from Invitrogen). To stimulate an inflammatory response, the THP-1 cells were treated with 100 ng/mL LPS.

### 4.2. Cell Proliferation Assay

For the cell proliferation assay, four culture groups were formed as follows: (1) the control group, in which the cells were cultured in low-glucose DMEM containing 10% FBS and 100 U penicillin/streptomycin; (2) the selenium group, containing 10% FBS, 100 U penicillin/streptomycin, and 5 ng/mL selenium; (3) the exosome group, containing 10% FBS, 100 U penicillin/streptomycin, and 5 μg/mL exosomes; and (4) the selenium exosome group, containing 10% FBS, 100 U penicillin/streptomycin, and 5 ng/mL selenium-stimulated exosomes. Briefly, the cells were plated at a density of 1 × 10^3^/well in a 96-well plate (BD Biosciences Pharmingen, San Diego, CA, USA). Cell proliferation was determined using a WST-based assay kit (EZ-Cytox, Daeil Lab, Seoul, Korea), according to the manufacturer’s protocol. Absorbance was measured at 450 nm using a microplate reader (Molecular Devices, San Jose, CA, USA).

### 4.3. Quantitative Reverse Transcription PCR (qRT-PCR)

Total RNA was extracted using RiboEx reagent (GeneAll, Seoul, Korea), according to the manufacturer’s instructions. The RNA was then reverse transcribed into cDNA using Maxime RT PreMix (iNtRON, Seongnam, Korea). To amplify the target genes, cDNA was mixed with specific primers. Real-time PCR was performed using LightCycler 480 SYBR Green I Master Mix (Roche Molecular Systems, Pleasanton, CA, USA). The experiments were performed in triplicate using the primers listed in Table S1. The values were normalized to those of glyceraldehyde-6-phosphate dehydrogenase (GAPDH), taken as an internal control, and the relative mRNA expression levels were calculated using the C_T_ method.

### 4.4. Exosome Isolation and Characterization

For exosome isolation, the cells were cultured at 1 × 10^7^ cells in a T175 flask (Nunc) with exosome-depleted FBS (Invitrogen). Specifically, the AdMSCs were pretreated with selenium at a final concentration of 5 ng/mL in exosome-depleted culture medium for 48 h. Exosomes were isolated from the cultured AdMSC medium using an exosome isolation kit (System Biosciences, Palo Alto, CA, USA) in accordance with the manufacturer’s instructions. Briefly, the culture medium was transferred to a fresh conical tube. After centrifugation at 1500× *g* for 5 min, the supernatant was transferred to another fresh conical tube, and ExoQuick-TC was added and mixed by inverting four times. The mixture was incubated overnight at 4 °C and centrifuged at 1500× *g* for 30 min on the following day. After removing the supernatant, the exosomes were resuspended in 100 μL PBS (Invitrogen) (volume ratio 1:100 (PBS:supernatant)). The isolated exosomes were quantified using a BCA protein assay kit (Invitrogen), and the final exosomes were stored at −80 °C for further experiments. To observe the shape of the exosomes, isolated exosomes were observed via transmission electron microscopy (TEM; JEM-1011, Jeol, Akishima, Japan). The size distribution of the exosomes was evaluated using a nanoparticle tracking system, according to the manufacturer’s instructions (NanoSight NS300, Malvern Panalytical, Malvern, UK).

### 4.5. Western Blotting

Western blotting was performed to evaluate the exosome markers. Briefly, the exosomal pellet was resuspended in 200 μL of RIPA buffer containing a phosphatase inhibitor cocktail, and the lysates were incubated at room temperature for 5 min after vortexing for 15 s. The concentrations were determined using a BCA assay kit (Invitrogen). The samples were boiled at 95 °C for approximately 5 min, and then chilled on ice for 5 min before being loaded onto the gel. The proteins were separated using a 12% sodium dodecyl sulfate-polyacrylamide gel and then transferred onto a polyvinylidene fluoride membrane (Bio-Rad Laboratories, Redmond, WA, USA). After blocking with 5% skim milk (BD Pharmingen, San Diego, CA, USA) for 1 h, the blots were incubated with indicated primary antibodies at 4 °C (exosome panel, Abcam #ab275018, Cambridge, UK), according to the manufacturer’s protocols. Subsequently, the blots were incubated with horseradish peroxidase-conjugated anti-rabbit secondary antibody (1:1000; GeneTex, Irvine, CA, USA) for 1 h at room temperature. The membrane was visualized using SuperSignal West Femto Maximum Sensitivity Substrate (Invitrogen), according to the manufacturer’s instructions. Finally, signals were detected using the LAS4000 system (GE Healthcare, Uppsala, Sweden).

### 4.6. ROS Analysis

Dihydroethidium (DHE, Invitrogen), an oxidative fluorescent dye, was used to detect superoxide, which binds to DNA in the nucleus. Briefly, cells cultured in a 12-well plate (Nunc) were treated with 10 μM DHE for 30 min at 37 °C in an incubator protected from light. The cells were washed with PBS and fixed with 4% paraformaldehyde (Biosesang, Seongnam, Korea). Fluorescent images were obtained using a fluorescence microscope (Olympus IX71, Tokyo, Japan).

### 4.7. Multiplex Supernatant Cytokine Assay

The culture supernatant was analyzed using a Magnetic Luminex Assay-human premixed multi-analyte kit (R&D Systems, Minneapolis, MN, USA), according to the manufacturer’s instructions. As described previously [26], 50 μL of the sample was added to 50 μL of the diluted microparticle cocktail. The mixture was then incubated for 2 h at room temperature on a shaker at 800 rpm. After washing with wash buffer thrice, 50 μL of diluted biotin-antibody cocktail was added to the wells and incubated for 1 h at room temperature on a shaker at 800 rpm. After four washes, 50 μL of diluted streptavidin-PE was added and incubated for 30 min at room temperature on a shaker at 800 rpm. After washing with the buffer, the resuspended microparticles were analyzed using a Luminex analyzer. All samples were analyzed in duplicate, and the concentrations were determined using an appropriate standard curve.

### 4.8. Tube Formation Assay

A Matrigel assay was performed to analyze capillary network formation. A Matrigel basement membrane matrix was purchased from Invitrogen. Briefly, 2 × 10^5^ HUVECs were seeded in 24-well plates (BD Falcon, Swedesboro, NJ, USA) after Matrigel-coating for 30 min in a 37 °C incubator. The cells were cultured in EGM-2 medium (Cambrex, Lonza, Walkersville, MD, USA) supplemented with 10% FBS and 1% P/S for 24 h. In vitro tube formation was evaluated using microscopy (Olympus).

### 4.9. In Vitro Wound Healing Assay

For the wound scratch assay, human dermal fibroblasts were plated into 12-well plates (BD Falcon) at a density of 2 × 10^5^ cells/well. The cells were cultured until they reached 100% confluence. The cells were scratched with a 1000 µL pipette tip to create an artificial wound. The scratch borders were immediately observed and photographed using a phase microscope. Damaged cells were incubated in the presence of 5 ng/mL selenium, 5 µg/mL exosomes, and 5 ng/mL selenium-stimulated 5 µg/mL exosomes for 24 h; the culture medium was used as a control. Cell migration was analyzed to determine the extent to which the cells had progressed from the scratch line. The assays were performed in triplicate, and cell migration was quantified using the ImageJ software (https://imagej.nih.gov/ij/, accessed on 15 November 2021).

### 4.10. In Vivo Wound Healing Assay

Male SPF-CrlOri:CD1(ICR) mice were used in this study. ChemOn Inc. (www.chemon.co.kr) at Yongin-si, Gyeonggi-do, Republic of Korea approved and performed all the procedures. Briefly, the animals (5 male donors from each group) were anesthetized by inhalation with isoflurane, hairs were removed using clippers, and wounds were induced using a biopsy punch (8 mm). The wound was covered with gauze and wrapped with an elastic bandage (Corban) to protect the wound area. The gauze and elastic bandages were re-dressed daily. The test article was administered subcutaneously three times (on the wound induction day, and on the 3rd and 7th days after induction). Clinical signs, body weight, wound area, and histological analysis were evaluated. The wound area was analyzed using image analysis software (Image Pro ver. 6.3) by taking pictures on the day of wound induction and 3, 7, and 14 days after induction. The mice were sacrificed on day 15 and skin samples were obtained for histology. Tissue samples were fixed with 10% formalin for 24 h and embedded in paraffin for hematoxylin and eosin (H&E) staining. The skin tissue, including the induced site, was stained with H&E, observed using an optical microscope (BX61, Olympus, Tokyo, Japan), and photographed (DP80, Olympus, Tokyo, Japan). The length and thickness of the newly formed neoepithelium by re-epithelialization at the wound site was measured as shown in the photo in reference [39]. In the wound area, the diameter of the granulation tissue and the thickness of the granulation tissue were measured from both wound edges formed by replacing the damaged skin in the scar tissue area, not the normal structure. Data were analyzed by Student’s *t*-test comparison procedures for comparison between the negative article administration group and the test article administration groups.

### 4.11. Library Preparation and Sequencing

After evaluating the RNA quality, it was quantified using a NanoDrop 2000 Spectrophotometer (Thermo Fisher Scientific, Waltham, MA, USA). For exosomal RNAs derived from AdMSCs and selenium-stimulated AdMSCs, the NEBNext Multiplex Small RNA Library Prep kit (New England BioLabs, Inc., Ipswich, MA, USA) was used to construct libraries according to the manufacturer’s guidelines. As described previously [26], 1 µg of total RNA from each sample was used to ligate the adaptors, and cDNA was synthesized using reverse transcriptase with specific primers. A PCR was performed for library amplification, and the libraries were purified using the QIAquick PCR Purification Kit (Qiagen, Inc., Hilden, Germany) and AMPure XP beads (Beckman Coulter). Yield and size distribution of the small RNA libraries were evaluated using an Agilent 2100 Bioanalyzer for the high-sensitivity DNA assay (Agilent Technologies, Santa Clara, CA, USA). High-throughput sequences were produced by the NextSeq 500 system via single-end sequencing (Illumina, San Diego, CA, USA). Sequence reads were mapped using the software tool, Bowtie2, to obtain a BAM file (alignment file). Mature miRNA sequences were used as a reference for mapping. Read counts mapped on the mature miRNA sequence were extracted from the alignment file using BEDtools (v2.25.0) and Bioconductor, which uses R (version 3.2.2) statistical programming language (R development Core Team, 2011). Read counts were used to determine miRNA expression levels. Quantile normalization was used for the comparison between samples, and miRWalk 2.0 was used for the miRNA target study.

### 4.12. Statistical Analysis

All values are expressed as mean ± standard deviation (SD). One-way analysis of variance (ANOVA) for data with >2 groups and Student’s *t*-test for paired group data were performed using GraphPad Prism (Prism ver. 7, Graph Pad Software, Inc., La Jolla, CA, USA) with Tukey corrections. Differences with *p* < 0.05 (*) and < 0.01 (**) were considered significant.

## Data Availability

All the data analyzed in this study are included in this published article. The data used to support the findings of this study are available from the corresponding author upon request.

## Supplementary Materials

The supporting information can be downloaded at: https://www.mdpi.com/article/10.3390/ijms231911543/s1.
