# Supplementary material for: Selenium-Stimulated Exosomes Enhance Wound Healing by Modulating Inflammation and Angiogenesis
Source: Int J Mol Sci. 2022 Sep 29;23(19):11543. doi: 10.3390/ijms231911543 (PMC9570007; doi:10.3390/ijms231911543)
Supplement: Supplementary file 1 [file ijms-23-11543-s001.zip › ijms-1924866-supplementary.pdf]

Supplementary information

Selenium-Stimulated Exosomes Enhance Wound Healing by Modulating Inflammation and Angiogenesis

June Seok Heo

Cell Therapy Center, Severance Hospital, Seoul 03722, Korea

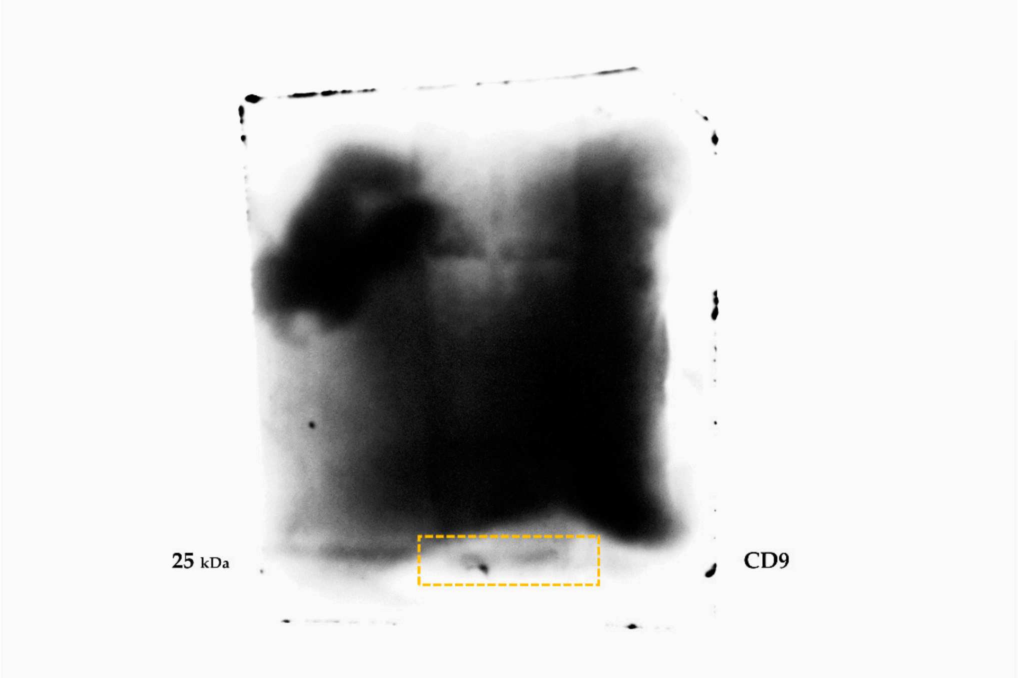
25 kDa
CD9

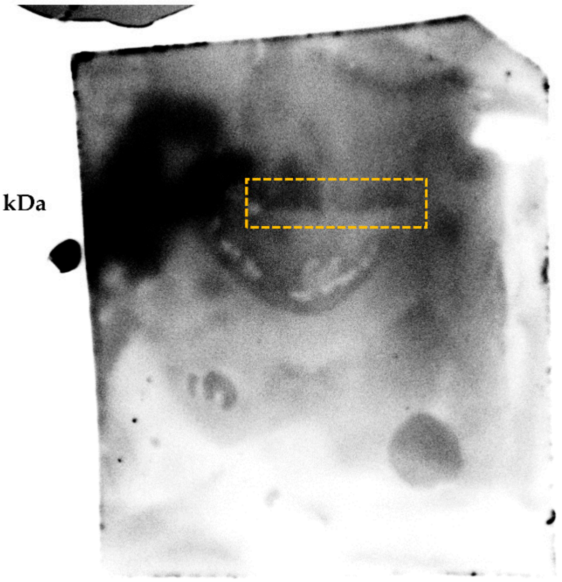
53 kDa
CD63

26 kDa

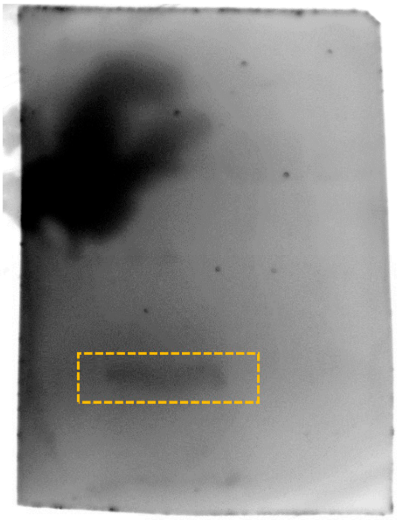

CD81

70 kDa

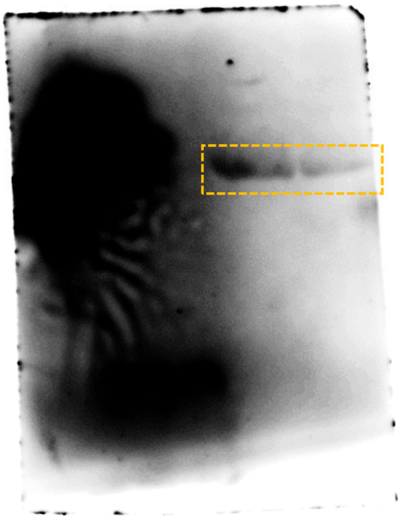

HSP70

Figure S1. CD9, CD63, CD81 and HSP70 of exosomal markers were detected in AdMSCs-derived exosomes by Western blot assay.

Table S1. Primers sequences used for real-time PCR.

Primer Name	Sequence
GAPDH	F: ACCCACTCCTCCACCTTTGA<br>R: CTGTTGCTGTAGCCAAATTCGT
IL-6	F: AGACAGCCACTCACCTCTTCAG<br>R: TTCTGCCAGTGCCTCTTTGCTG
IL-8	F: AAGAGAGCTCTGTCTGGACC<br>R: GATATTCTCTTGGCCCTTGG
TSG-6	F: GGTGTGTACCACAGAGAAGCA<br>R: GGGTTGTAGCAATAGGCATCC
TGF-ß1	F: TACCTGAACCCGTGTTGCTCTC<br>R: GTTGCTGAGGTATCGCCAGGAA
Runx2	F: TTGCAGCCATAAGAGGGTAG<br>R: GTCACTTTCTTGGAGCAGGA
TNFα	F: TTGAGGGTTTGCTACAACATGGG<br>R: GCTGCACTTTGGAGTGATCG
Arginase-1	F: ACAGTTTGGCAATTGGAAGCA<br>R: CACCCAGATGACTCCAAGATCAG
CD206<br>(Mannose receptor)	F: TTCGGACACCCATCGGAATTT<br>R: CACAAGCGCTGCGTGGAT
Type I collagen	F: GTCACCCACCGACCAAGAAACC<br>R: AAGTCCAGGCTGTCCAGGGATG
Type III collagen	F: GGAGCTGGCTACTTCTCGC<br>R: GGGAACATCCTCCTTCAACAG
BMP7	F: CCAACGTCATCCTGAAGAAATAC<br>R: GCTTGTAGGATCTTGTTCATTGG
C/EBPA	F: CCAAGAAGTCGGTGGACAAGAA<br>R: TCATTGTCACTGGTCAGCTCCA
IL-10	F: TCTCCGAGATGCCTTCAGCAGA<br>R: TCAGACAAGGCTTGGCAACCCA
p16	F: CGAATAGTTACGGTCGGAGG<br>R: GCATGGTTACTGCCTCTGGT

p21	F: GCGATGGAACTTCGACTTTG<br>R: CGTTTTCGACCCTGAGAGAGTC
CD163	F: CGGCTGCCTCCACCTCTAAGT<br>R: ATGAAGATGCTGGCGTGACA
Angiopoietin1<br>(ANGPT1)	F: CTTCAAGGCTTGGTTACTCGTC<br>R: CTCTTCCTCTCTTTTTCCTCCC
KDR<br>(Flk-1)	F: AACGACTGCCTTATGATGCC<br>R: ACTGTCCTGCAAGTTGCTGTC
Vasohibin1<br>(VASH1)	F: CTCAAGATTGGCAAAGGGACG<br>R: TTTCACTGCGGCTGTTCCTGC
VEGF	F: AGCCTTGTTCAGAGCGGAGAA<br>R: TAACTCAAGCTGCCTCGCCTT
αSMA	F: ACTGAGCGTGGCTATTCCTCCGTT<br>R: GCAGTGGCCATCTCATTTTCA
Elastin	F: GGCCATTCCTGGTGGAGTTCC<br>R: AACTGGCTTAAGAGGTTTGCCTCCA

Table S2. Upregulation of miRNAs in exosomes secreted by AMSCs.

	Gained miRNAs	Sel-Exos/Exos
Anti-Inflammatory	miR-146a-5p	94.612
	miR-340-5p	38.002
	miR-223-3p	34.601
	miR-125b-5p	19.029
	miR-16-5p	12.443
	miR-149-3p	12.443
	miR-105-5p	11.715
	miR-181c-3p	11.715
	miR-146b-5p	4.767
	miR-181a-5p	3.795
Pro-Angiogenic	miR-378a-3p	2.546
Proliferation/Migration	miR-205-5p	118.861
	miR-146a-5p	94.612
	miR-184	3.814
Remodeling	miR-129-5p	1202.524
	miR-29b-3p	594.584

Table S3. Downregulation of miRNAs in exosomes secreted by AMSCs.

	Lost miRNAs	Sel-Exos/Exos
Pro-Inflammatory	miR-140-5p	0.002
	miR-33a-5p	0.072
	miR-155-5p	0.403
Anti-Angiogenic	miR-20a-5p	0.001
	miR-192-5p	0.107
	miR-24-3p	0.292
	miR-320b	0.305
Anti-Proliferation/Migration	miR-99b-3p	0.00007
	miR-185-5p	0.031
	miR-141-3p	0.351

Figure S2. Gene ontology enrichment of the top 10 upregulated miRNAs.

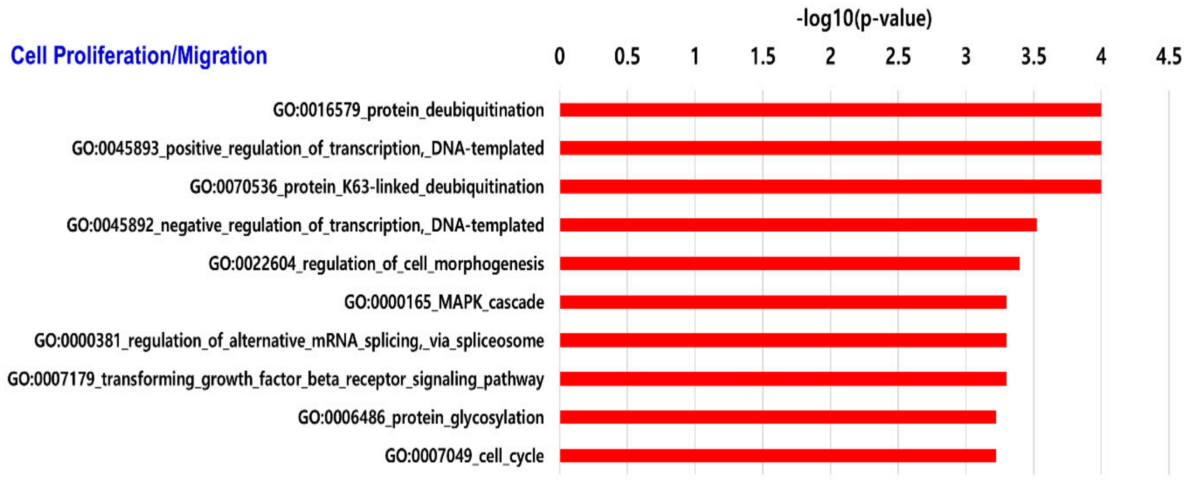

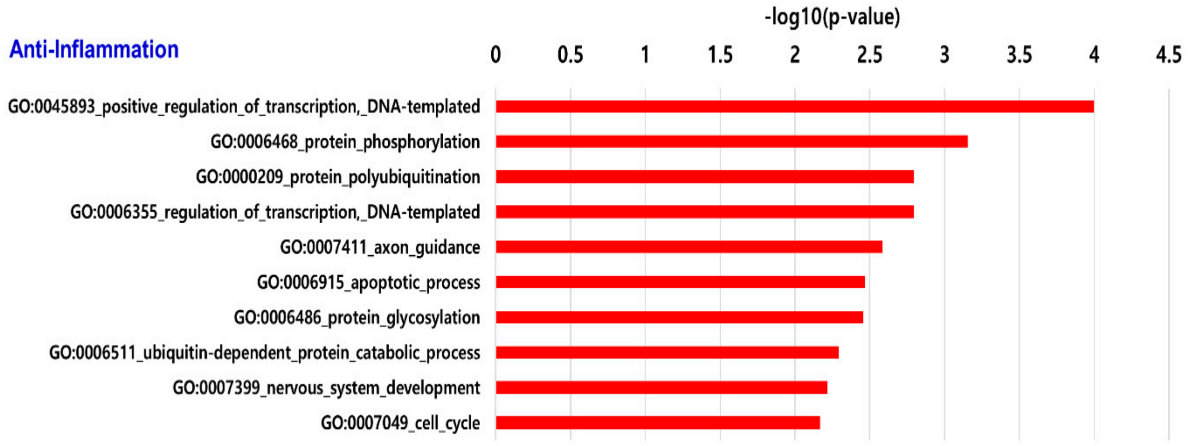

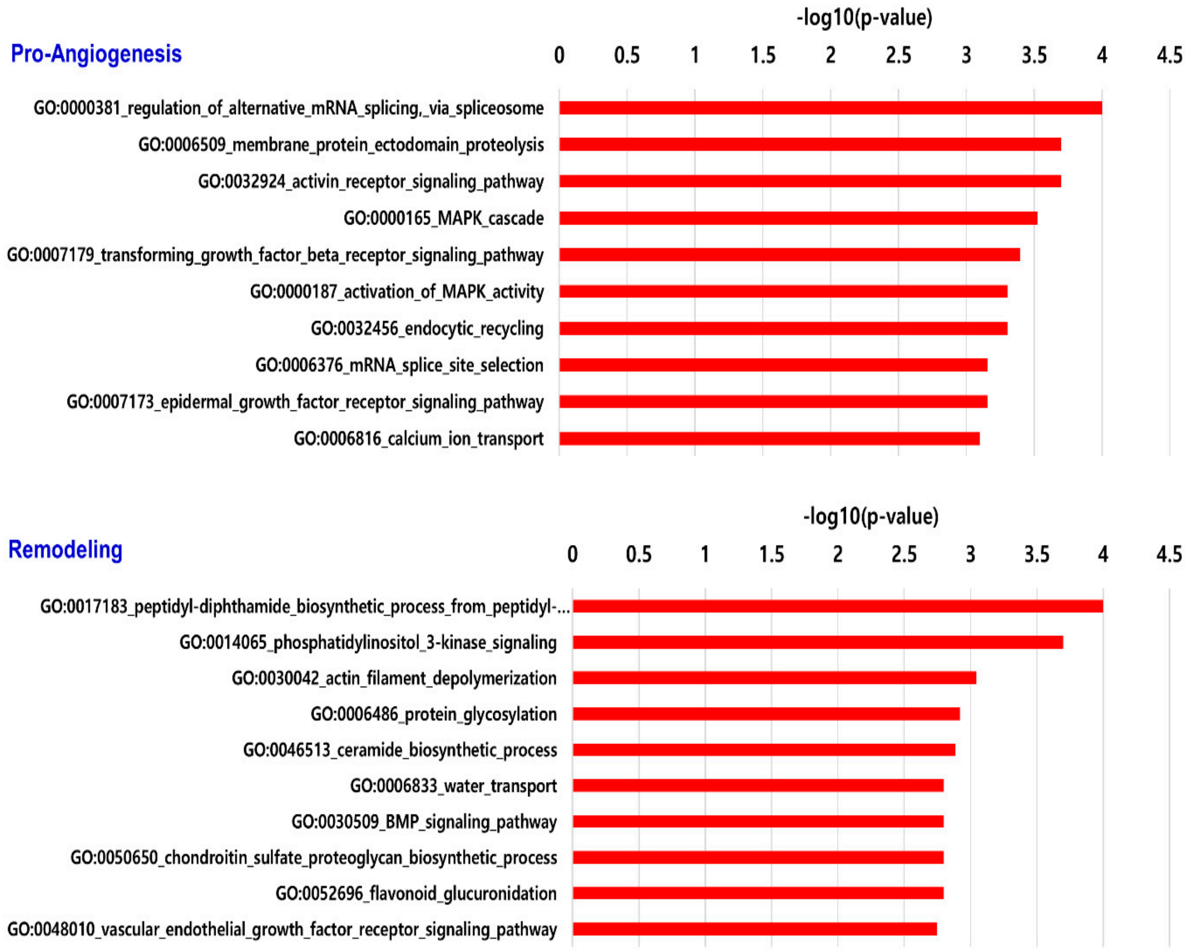

Pro-Angiogenesis
-log10(p-value)
0
0.5
1
1.5
2
2.5
3
3.5
4
4.5
GO:0000381_regulation_of_alternative_mRNA_splicing,_via_spliceosome
GO:0006509_membrane_protein_ectodomain_proteolysis
GO:0032924_activin_receptor_signaling_pathway
GO:0000165_MAPK_cascade
GO:0007179_transforming_growth_factor_beta_receptor_signaling_pathway
GO:0000187_activation_of_MAPK_activity
GO:0032456_endocytic_recycling
GO:0006376_mRNA_splice_site_selection
GO:0007173_epidermal_growth_factor_receptor_signaling_pathway
GO:0006816_calcium_ion_transport
Remodeling
-log10(p-value)
0
0.5
1
1.5
2
2.5
3
3.5
4
4.5
GO:0017183_peptidyl-diphthamide_biosynthetic_process_from_peptidyl-...
GO:0014065_phosphatidylinositol_3-kinase_signaling
GO:0030042_actin_filament_depolymerization
GO:0006486_protein_glycosylation
GO:0046513_ceramide_biosynthetic_process
GO:0006833_water_transport
GO:0030509_BMP_signaling_pathway
GO:0050650_chondroitin_sulfate_proteoglycan_biosynthetic_process
GO:0052696_flavonoid_glucuronidation
GO:0048010_vascular_endothelial_growth_factor_receptor_signaling_pathway
